# Early prediction of cerebral malaria by ^1^H NMR based metabolomics

**DOI:** 10.1186/s12936-016-1256-z

**Published:** 2016-04-12

**Authors:** Soumita Ghosh, Arjun Sengupta, Shobhona Sharma, Haripalsingh M. Sonawat

**Affiliations:** Department of Chemical Sciences, Tata Institute of Fundamental Research, 1-Homi Bhabha Road, Mumbai, 400 005 India; Department of Biological Sciences, Tata Institute of Fundamental Research, 1-Homi Bhabha Road, Mumbai, 400 005 India

**Keywords:** Cerebral malaria, NMR, Prediction, Metabolomics, OPLS-DA, Lipids

## Abstract

**Background:**

Cerebral malaria (CM) is a life-threatening disease, caused mainly by *Plasmodium falciparum* in humans. In adults only 1–2 % of *P. falciparum*-infected hosts transit to the cerebral form of the disease while most exhibit non-cerebral malaria (NCM). The perturbed metabolic pathways of CM and NCM have been reported. Early marker(s) of CM is(are) not known and by the time a patient exhibits the pathological symptoms of CM, the disease has progressed. Murine CM, like the human disease, is difficult to assign to specific animals at early stage and hence the challenge to treat CM at pre-clinical stage of the disease. This is the first report of prediction of CM in mice using a novel strategy based on ^1^H nuclear magnetic resonance (NMR)-based metabolomics.

**Methods:**

Mice were infected with malarial parasites, and serum was collected from all the animals (CM/NCM) before CM symptoms were apparent. The assignment of mice as NCM/CM at an early time point is based on their symptoms at days 8–9 post-infection (pi). The serum samples were subjected to ^1^H NMR-based metabolomics. ^1^H NMR spectra of the serum samples, collected at various time points (pi) in multiple sets of experiments, were subjected to multivariate analyses.

**Results:**

The results from orthogonal partial least square discriminant analyses (OPLS-DA) suggest that the animals with CM start to diverge out in metabolic profile and were distinct on day 4 pi, although by physical observation they were indistinguishable from the NCM. The metabolites that appeared to contribute to this distinction were serum lipids and lipoproteins, and 14–19 % enhancement was observed in mice afflicted with CM. A cut-off of 14 % change of total lipoproteins in serum predicts 54–71 % CM in different experiments at day 4 pi.

**Conclusion:**

This study clearly demonstrates the possibility of differentiating and identifying animals with CM at an early, pre-clinical stage. The strategy, based on metabolite profile of serum, tested with different batches of animals in both the sex and across different times of the year, is found to be robust. This is the first such study of pre-clinical prognosis of CM.

**Electronic supplementary material:**

The online version of this article (doi:10.1186/s12936-016-1256-z) contains supplementary material, which is available to authorized users.

## Background

Pathological manifestations of malaria encompass a wide range of disease syndrome, from uncomplicated malaria to severe life-threatening forms of malaria. Cerebral malaria (CM) is a potentially fatal form of malaria caused by the human malaria parasite *Plasmodium falciparum*, which causes the death of several thousand children per year in sub-Saharan Africa [1]. Not all individuals infected with *P. falciparum* succumb to severe malaria and only about 1–2 % of infected hosts succumb to the cerebral form of the disease [2, 3]. With the advent of current anti-malarial treatments, the chances of survival of CM cases have increased substantially, but it is observed that survivors may suffer from long-term neurological deficits [1, 4]. Historically, the presence of parasites in comatose patients in malaria-endemic areas was considered adequate reason for the administration of anti-malarials. The diagnosis of CM is difficult because coma could be a consequence of a variety of neurological syndromes. Often autopsy results would conclusively diagnose the disease, by which time it is too late for the patient. Beyond coma and other non-specific neurological symptoms, perhaps retinopathy is the only advancement that has become one of the best diagnostic indicators of CM [5]. The causes of CM are broadly ascribed to inflammation, sequestration and vascular blockage, leading to hypoxia and hypoglycaemia, resulting in complex neurological sequelae [6]. The susceptibility of an individual to a severe form of malaria, especially that of CM, has been the subject of study over several decades, so that such pathology may be prevented. However, as of now, no reliable predictor(s), clinical or molecular, are known that would be prognostic of CM at an early time point (pre-clinical or uncomplicated stages) in a malaria-infected individual. There is a need to develop tools and techniques that may provide an early indication of the susceptibility to the cerebral stage of the disease. Observations on human CM are possible only in *post*-*mortem* tissues, and at best in the cerebro-spinal fluids, but a longitudinal study is not feasible. Amongst animal models, the best available is the murine model, which may have certain limitations, but nevertheless exhibits a range of similarities, including perturbations in the integrity of the blood–brain barrier, infected red blood cell (RBC) sequestration to the cerebral microvasculature, microvascular damage, and generation of cognitive impairments [7–9].

The difficulties of a longitudinal study in humans are complicated by the genetic diversity of hosts and parasites, as are variation in the environment, and metabolic and physiological parameters. The cumulative incidence of CM is variable in mice. It ranges from 50 to 100 % [10]. In this laboratory setting, the partitioning of the C57Bl/6 mice infected with *Plasmodium berghei* ANKA strain into CM and non-cerebral malaria (NCM) is nearly 50 % [11] and this allowed the addressal of metabolic susceptibility not only within the same genotypes, but also within the same batch of animals. Clinical features, such as rectal temperature, the clinical manifestations of CM, such as ataxia, paralysis, coma, and convulsions, and a differential survival rate, makes the final classification of the disease into two definite classes of CM and NCM mice possible [11]. However, these CM symptoms are observable only at the late stages of the disease while in the early stages the animals are physiologically indistinguishable. Thus, it becomes possible to follow the same animal cohort as they move into two distinct groups and determine the metabolic differences.

CM-susceptible mice have been compared with NCM mice at the late stage of the disease [12–14]. The difference in the metabolic pathways in CM and NCM in various tissues and biofluids were delineated [13]. However, information and understanding of the early metabolic changes are lacking. Moreover, the methods that can predict CM are limited. The SmithKline Beecham, Harwell, Imperial College, Royal London Hospital, phenotype assessment (SHIRPA) protocol relates behavioural aspects of the mice to death [15, 16]. The method involves 40 independent parameters to be looked at simultaneously, which is difficult to apply in a high-throughput study. Furthermore, the strategy is difficult to translate to humans because the tests are specific to rodents. The chemokines, CXCL4 and CXCL10 levels also predict the risk of CM in human [17]. This prediction is, however, based on data collected from patients who have already succumbed to CM. Similarly, malaria score for adults (MSA) predicts deaths in adults but this is again based on parameters of patients after they succumbed to the disease [18]. In another study, papilloedema and extramacular retinal oedema were used to predict death in CM [19]. These cases were also the advanced stages of CM. It has been shown that the angiopoietin 1 and 2 were associated with CM. However, even in this study the patients had already succumbed to the disease [20]. Thus, there is no reliable predictor of the disease at an early time point.

With the advent of ‘metabolomics’ [21], it is possible to build predictive models of metabolites that would discriminate CM from NCM during the sub-clinical stages. Metabolomics is a hypothesis-free study, which can be used to assay the observable metabolites in biofluids/tissue, develop temporal trajectories of the metabolites, and thus follow the disease progression and identify metabolic marker/s for specific stages of the disease. One of the objectives of this investigation was to identify early metabolic signature(s) of CM, using serum samples. In this paper the results of these investigations are presented. The results suggest that serum lipids/lipoproteins levels may represent early indicators of the onset of CM in both male and female animals. Temporal tracking of the CM and NCM animals showed that serum lipid/lipoprotein levels are higher in CM with respect to NCM in early days of infection.

## Methods

### Animals used for the study

**Fig. 1:**
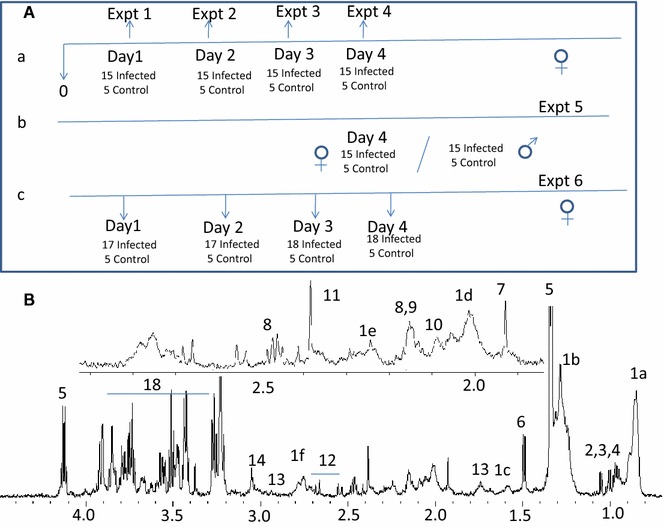
The scheme of retro-orbital bleeding from the group of mice in a set of different experiments. The *down arrows* refer to the early time point in which mice were bled. **A** Scheme for retro-orbital bleeding in different experiments. **B** Typical 700 MHz ^1^H NMR spectrum of serum of a control C57Bl/6. *1* Lipids [*1a* –CH_3_, *1b* –(CH_2_)_n_–, *1c* –CH_2_CH_2_CO, *1d* –CH_2_C=C, *1e* –CH_2_=CO–, *1f* =C–(CH_2_)–C=], *2* leucine, *3* isoleucine, *4* valine, *5* lactate, *6* alanine, *7* acetate, *8* glutamine, *9* glutamate, *10* methionine, *11* acetate, *12* citrate, *13* lysine, *14* creatine, *15* choline, *16* phosphocholine, *17* GPC, *18* glucose

The animals were treated in accordance with the guidelines set forth by the Institutional Animal Ethics Committee of TIFR (IAEC approval no: TIFR/IAEC/2010-3). The mice used for the study were C57Bl/6 of 6–8 weeks and weighing 20–25 g. They were maintained in 12 h light and dark cycle at 22 ± 2 °C and had free access to water and standard food pellets. Initially, four separate experiments for female mice were performed with 20 mice in each experiment (Fig. 1). Blood was withdrawn from animals on different days post-infection (pi): day 1, day 2, day 3, and day 4 pi, in four separate experiments with different sets of mice, respectively. These experiments were done to understand the maximal difference in CM and NCM animals before symptoms of the disease are evident. Since the results show a maximal difference in the metabolic profile of serum in day 4 pi, the experiment for day 4 pi was repeated where the blood was withdrawn only at day 4 pi from a new set of animals. A separate experiment was conducted with a group of male mice to unravel sexual dimorphism in the molecular marker/s of CM. Yet another independent experiment was carried out to compare the metabolic profiles and to understand the sequence of events in the animals with CM/NCM. In order to understand the disease progression in CM versus NCM, mice were bled at day 3, 4 and 5 pi and the two groups of animals were compared in one experimental set-up. However, the mice used to compare the metabolic profile at day 3, 4 and 5 pi were different to avoid stress in mice due to consecutive daily bleeding.

### Inoculation of mice, disease progression and assessment

In each of the four experiments, five mice were used as uninfected controls. The remaining animals were injected intraperitoneally with 10^7^ RBCs infected with *P. berghei* ANKA [22]. Rectal temperature of the mice was measured daily using a digital thermometer. The infected animals were monitored for the neurological symptoms of CM from day 5 pi. Mice were considered to have CM if they had rectal temperature <34 °C accompanied with neurological symptoms such as ataxia, convulsions and coma. The NCM mice, on the other hand, exhibited no neurological symptoms and had body temperature >34 °C. At day 8/9 pi the symptoms of CM were clearly visible in addition to the reduced rectal temperature which helped in categorizing the mice into CM and NCM. Thus, categorization of animals as having CM or NCM was unambiguous. Some of the mice died during the night and some had borderline rectal temperature at day 9 pi. These were excluded from the study. Blood (100 µl) was collected by retro-orbital bleeding from the mice at indicated time points pi in the four schemes (Fig. 1). The assignment of the sample (blood) to CM and NCM mice at an earlier time point is based on the diseased state of the mice at day 9 pi.

### Sample preparation for ^1^H nuclear magnetic resonance (NMR) of sera

The blood samples were incubated at 37 °C for 10 min, centrifuged for 10 min at 13,100*g* at 4 °C. Supernatant was collected, frozen immediately in liquid N_2_ and stored at −80 °C until the nuclear magnetic resonance (NMR) experiment. For NMR experiments, 60 µl of sample was mixed with 500 µL of buffer (0.075 M Na_2_HPO_4_·7H_2_O, 4 % NaN_3_, 0.02 % TSP, pH 7.4). The buffer recipe was provided by Bruker Biospin, Metabonomic unit, Rheinstetten, Germany.

### ^1^H NMR of sera

^1^H NMR spectra at 310 K was acquired on AVANCE III 700 MHz Bruker spectrometer with triple resonance inverse probe using D_2_O (10 % of the sample volume) as the field-frequency lock. The samples were placed in 5-mm NMR tubes. The pulse sequence used for the experiment was of the form—RD-90°-(τ-180-τ-)n-90°-ACQ. Relaxation delay (RD) of 4 s was used between consecutive transients. Water signal was suppressed by continuous irradiation using low power pulse during RD. Car–Purcell–Meiboom–Gill (CPMG) spin echo block (τ-180-τ-)n with a loop count (n) of 128 was used to attenuate the broad signals from the macromolecules. Echo time for the experiment (τ) was 300 µs. In these experiments 32 transients were collected into 88,640 data points using a spectral width of 20.06 ppm. The 90° pulse lengths were individually determined for all the samples. The free induction decays were subjected to exponential multiplication leading to an additional line broadening of 1 Hz, a Gaussian multiplication of 0.01 Hz, and a *sine*-squared, bell apodization function prior to Fourier transformation. The resulting spectra were phased and baseline corrected by the automation programs provided by Bruker Biospin. The assignments of metabolites were based on 2D-COSY, 2D-(^1^H–^1^H) TOCSY and 2D J-resolved spectroscopy experiments. The assignment was further aided using Human Metabolome Database (HMDB) [23] and also cross-checked from literature [24]. For COSY and TOCSY experiments, 64 transients per increment and 256 increments were collected in the indirect dimension. A QSINE function with 2048 and 1024 digital points was used for processing. An exponential multiplication of 0.20 and 0.30 Hz in the direct and indirect dimension was used, respectively. TOCSY was processed using *sine* function. An exponential multiplication of 0.20 and 0.30 Hz in the direct and indirect dimension was used, respectively. In addition, a Gaussian multiplication of 0.1 Hz in the indirect dimension was applied for the TOCSY. 2D J-resolved spectra were also recorded to aid the assignments of the metabolites. The pulse sequence is of the form—RD-90-t1-180-t1-ACQ. The RD was 2 s and the number of scans being one for all experiments. The number of data points in direct and indirect dimension was 16k and 256, respectively. The spectral widths in direct and indirect dimensions were 16.63 ppm and 0.15 Hz ppm, respectively. After the Fourier transformation, baseline correction was performed followed by 45° tilt of the spectrum.

### Data pre-processing and statistical analysis

The spectral region of 0.5–4.2 ppm is bucketed into frequency windows of 0.003 ppm and was normalized with respect to the working region. The region corresponding to water (4.5–5.5 ppm) was excluded during binning to avoid artefacts due to water pre-saturation. The region corresponding to aromatic regions was also excluded because it had poor signal to noise ratio compared to the aliphatic region. All the binning and normalizations are performed in AMIX 2.0 (Bruker Biospin). The resulting matrix was imported in SIMCA P+ 12.0 (Umetrics AB, Malmo, Sweden) for multivariate analysis.

### Principal component analysis (PCA) and orthogonal partial least square discriminant analysis (OPLS-DA)

The 2D data matrix obtained after normalization is subjected to PCA analysis. PCA was done to look into trend of the data and to check if there were outliers. This was followed by OPLS/OPLS-DA [25]. OPLS-DA gives segregation between two classes along predictive component. The R^2^X and Q^2^Y are the two parameters that judge the model. While the former explains the total explained variation in the data, the latter is a cross-validation parameter that explains the predictive power of the model. One-seventh of the data are excluded in each cross-validation round. The metabolites that are responsible for segregation are represented by OPLS-DA coefficients plots [26]. The plots are generated using GNUPLOT.

## Results

### Early metabolic markers in serum for CM

**Fig. 2:**
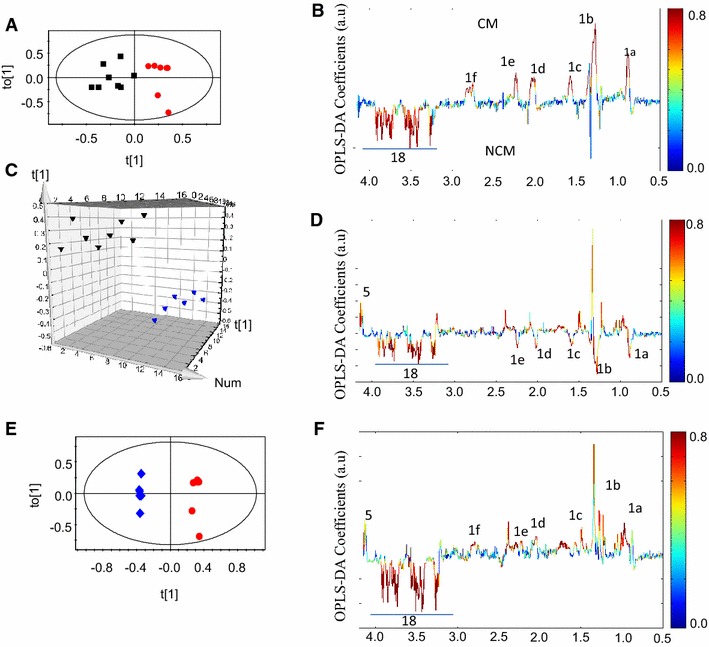
Pairwise OPLS-DA scores and coefficients plot of ^1^H NMR spectra of serum of CM, NCM and control at day 4 post-infection in experiment 4. **A** OPLS-DA scores plot of CM and NCM at day 4 post infection, **B** OPLS-DA coefficient plot of **A**, **C** OPLS-DA scores plot of NCM and control, **D** OPLS-DA coefficient plot of **C**, **E** OPLS-DA scores plot CM and control, **F** OPLS-DA coefficient plot of **E**. The *red*, *black* and *blue symbols* denote CM, NCM and control, respectively. The *ellipse* in the scores plot is a 95 % Hotelling T^2^. The *colour bar* indicates the correlation of the metabolites in the segregation between two concerned classes

**Fig. 3:**
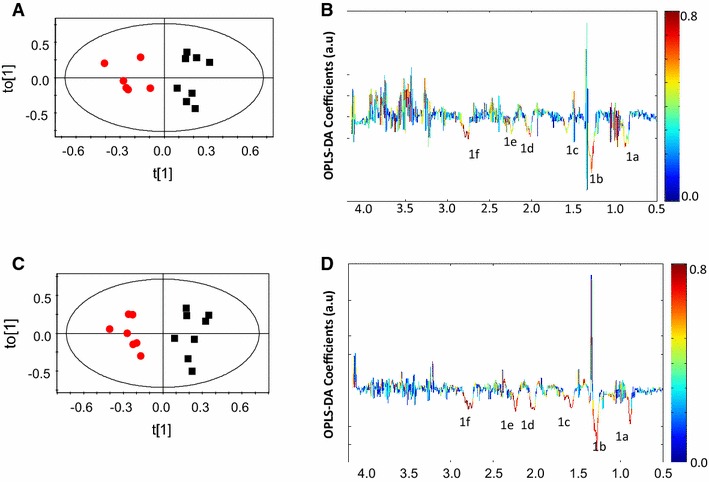
OPLS-DA scores and coefficient plot of CM and NCM at day 4 post infection for experiment 5. **A** OPLSDA scores plot of CM and NCM for female mice, **B** OPLS-DA coefficient plot of **A**, **C** OPLSDA scores plot of CM and NCM for male mice, **D** OPLS-DA coefficient plot of **C**. The *red* and *black symbols* denote CM and NCM, respectively. The *ellipse* in the scores plot is a 95 % Hotelling T^2^. The *colour bar* indicates the correlation of the metabolites in the segregation between two concerned classes

The scheme for blood collection from mice in different experiments is depicted in Fig. 1A. The ^1^H NMR of serum of an uninfected control mouse was assigned using COSY and is shown in Fig. 1B. All the discriminant analyses involving CM and NCM at days 1–5 were based on their physiological status at days 8–9 pi. The OPLS-DA models of the ^1^H NMR spectra of the sera samples of CM and NCM have poor segregation on day 1 and day 2 pi (Table 1). On day 3 pi, the segregation between the classes increases compared to day 1 and day 2 pi as is evident from the increased value of Q^2^Y of the corresponding OPLS-DA model. On day 4 pi, further improvement in segregation between CM and NCM was evident from the high Q^2^Y value (Fig. 2A; Table 1). The molecules that contribute to the segregation are glucose, moieties of lipids and lipoproteins, such as –CH_3_, –(CH_2_)n–, –CH_2_CH_2_CO, –CH_2_C=C, –CH_2_C=O, =C–(CH_2_)–C= (Fig. 2B). The OPLS-DA coefficient plot (Fig. 2B) clearly suggests that lipids/lipoproteins concentrations are high in CM, while that of glucose is low. In addition, glycerophosphocholine (GPC) is high in NCM sera. The unsupervised PCA model was also able to segregate CM and NCM at day 4 pi (Additional file 1). Further separate statistical models were built with individual infected groups and controls. It was evident from such models that lipids are increased in the CM and decreased in NCM with respect to the controls (Fig. 2C–F). The predictive metabolite markers of CM and the robustness of such markers on day 4 pi were further investigated in another experiment which included a group of male and female mice. As seen in Table 1 and in Fig. 3A, the segregation of the ^1^H NMR profile of CM and the NCM are distinct. This distinction is due to the presence of a high concentration of serum lipids/lipoproteins in CM (Fig. 3B). Glucose is not involved in the segregation of CM and NCM. In order to make analysis independent of the terminal time point, a separate analysis was performed where the bins corresponding to lipids were denoted as the ‘response variables’. Thus, a separate OPLS model for day 4 pi was created to see if the groups segregate solely on the basis of lipids (1.54–1.62, 2.81–2.73, 2.24–2.31, 1.92–2.07, 0.82–0.89, 1.23–1.30 ppm). It is seen that in the case of the first experiment there are two mis-classifications of CM and in the second case there is mis-classification of one CM with a prediction rate of 71 and 83.3 % (Additional file 2a, b). In order to know whether the molecules contributing towards the segregation are the same in the other sex, ^1^H NMR spectrum of NCM and CM male mice were compared on day 4 pi with the help of multivariate statistics. It is clear (Fig. 3C) that the two groups of animals are indeed distinct and that serum lipids/lipoproteins are responsible for the segregation (Fig. 3D). Also, as in the case of the females, glucose appeared to have no significant contribution towards segregation (Fig. 3D). Moreover, when each of the infected class was compared with the controls it was found that lipids/lipoproteins increased in concentration in the serum of mice with CM while it is decreased in NCM (Additional file 3).

**Table 1:** Parameters of the OPLS-DA models created with ^1^H NMR profile of sera of CM and NCM for the scheme given in Fig. 1 and the repeat experiments

CM versus NCM	R^2^Y	Q^2^Y
Day 1 female	0.27	0.00
Day 2 female	0.39	0.14
Day 3 female	0.72	0.44
Day 4 female	0.84	0.70
Day 4 female (repeat)	0.87	0.54
Day 4 male	0.90	0.61

### Temporal tracking of CM and NCM mice

**Fig. 4:**
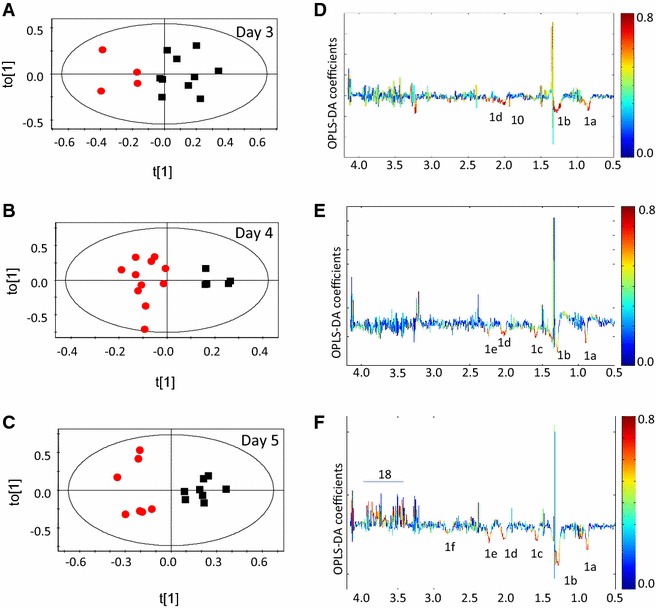
OPLS-DA scores and co-efficient plot of CM and NCM at different day post infection for experiment 6. **A** Scores plot of CM and NCM at day 3 post infection, **B** OPLS-DA coefficient plot of **A**, **C** scores plot of CM and NCM at day 4 post infection, **D** OPLS-DA coefficient plot of **C**, **E** scores plot of CM and NCM at day 5 post infection, **F** OPLS-DA coefficient plot of **E**. The *red* and *black symbols* denote CM and NCM, respectively. The *ellipse* in the scores plot is a 95 % Hotelling T^2^. The *colour bar* indicates the correlation of the metabolites in the segregation between two concerned classes

An important aspect of this study was to compare the metabolic profiles of animals with NCM and CM as the disease progresses. This is expected to provide useful information about the evolving metabolic status of the host. The results of the experiments mentioned so far involved collection of serum in separate sets of animals, of different batches, injected with parasites at different times of the year. A direct comparison of the profiles of the same set of mice with NCM/CM was not possible. Therefore, an independent experiment was set up to accomplish this. The parasitemia profile and survival is shown in Additional file 4. Serum samples were drawn on days 2, 3, 4, and 5 pi (Fig. 1), and PCA analysis of ^1^H NMR spectra for CM and NCM mice was performed. The analysis shows that at day 4 pi the CM mice form a separate clustering with respect to NCM (Additional file 5). This is followed by OPLS-DA analysis. The statistical models were created for NCM and CM (Table 2). The results clearly show that day 3 pi onwards, the serum profiles of NCM were distinct from CM (Fig. 4A–C). Serum lipids/lipoproteins significantly contribute towards segregation (Fig. 4D–F) and remain consistently high in CM sera compared to the NCM during the pre-clinical stage. The number of serum lipid/lipoprotein peaks increase in CM mice with respect to NCM mice as the disease progresses. The OPLS-DA models created for infected groups (CM/NCM) versus control are shown in Additional file 6a–c. In comparison to the uninfected control the serum lipoprotein is higher in CM on days 3–5 pi. The enhancement ranges from 14 ± 0.02 to 19 ± 0.09 % at day 4 pi in three independent experiments. A cut-off of fold change of 14 % of total lipoproteins in serum predicts 54–71 % CM. The change in the level of lipids in CM at day 4 pi is presented in (Additional file 7) for all the experiments. A ROC curve was plotted with the cross-validated ‘Y’ variables for the OPLS-DA plots created at day 4 pi (Additional file 8). The area under curve of the ROC plots are 1, 0.95 and 1, respectively, for day 4 pi of experiments 4, 5 and 6 of CM and NCM mice.

**Table 2:** Parameters of the OPLS-DA models created with ^1^H NMR profile of sera of CM and NCM for the scheme given in Additional file 3

NCM versus CM	R^2^Y	Q^2^Y
Day 2	0.00	0.00
Day 3	0.70	0.37
Day 4	0.87	0.62
Day 5	0.88	0.77

## Discussion

This study reports on the possible early molecular signatures that distinguish mice susceptible to CM versus those that are not, in the murine CM model of *P. berghei* ANKA parasite and C57Bl/6 mice. The earliest that the mice can be distinctly clustered is day 3 or 4 pi, the latter day being more robust (Q^2^ > 0.5). Mice with CM could be distinguished from NCM and control on the basis of higher levels of serum lipids/lipoproteins. Earlier, it has been established that at day 8–9 pi there is an increase in lipids/lipoproteins in the sera of CM mice [13]. It is now apparent that such increase starts from day 3 pi. This observation was robust and was seen in male mice as well. Thus, a 14–19 % increase of serum lipids/lipoproteins could be used as a marker for CM in mice. These results are obtained from a murine model of the disease wherein well-established mouse and parasite strains have been used, and the experiment including the number of parasites injected and the housing of animals are all tightly controlled. It should be noted that the distinction of CM from NCM is not batch/parasite/climate dependent. The result obtained here is robust not only in females but in the case of males as well.

It would be interesting to see if the change in lipids/lipoproteins observed here is robust enough to be seen in a field situation of human malaria. Since humans exhibit large variations in terms of age, ethnicity, lifestyles, etc., this might involve screening of large (entire) populations to arrive at the basal levels of circulating lipids/lipoproteins.

The temporal tracking throws light on the alterations in the host. It is important to note that serum glucose does not appear to play any discriminatory role in CM versus NCM at days 3–4 pi although on day 5 enhanced level of glucose is observed in NCM sera compared to CM. Moreover, serum lactate does not contribute significantly towards segregation of CM and NCM during this period. Thus, glycolysis, its substrate glucose and the end product lactate are not involved in differentiating CM and NCM animals at an earlier stage of infection but later time points and glucose levels indeed seem to be perturbed in CM in comparison with NCM. The possible formation of triglycerides from glucose at day 8–9 pi in CM has already been reported. In this study it seems that such a perturbation is operative from day 5 pi. The negative correlation of blood glucose and triglycerides in OPLS-DA plot at day 5 pi is suggestive of such perturbation.

Phospholipids are an important class of lipids which are required by the parasite during proliferation [27], are used for metabolic co-factor and membrane formation and affect host physiology [28, 29]. A sixfold increase in the phospholipids in the infected erythrocytes has been reported [30]. Parasites scavenge fatty acids from the host cells and serum, modify them by elongases and desaturases and incorporate them into membrane glycerides [31]. Thus, lipids of the host are channelized to various pathways in the parasites for their survival. The parasites can uptake host fatty acids [32, 33] and lipoproteins [34]. In cultured *P. falciparum*, large quantities of lipids are taken up during trophozoite and early schizont stages [35]. This parasite-mediated uptake of lipids/lipoproteins could alter the host-circulating homeostasis, which in turn could affect the level of lipids/lipoproteins in the host serum. One of the characteristics of CM is sequestration of parasites [36] and increase in the levels of TNF [37]. Both these parameters have an effect on the lipid metabolism, which might cause an elevation in the levels of lipids/lipoproteins, specifically in CM. All three steps of lipid metabolism, i.e., fatty acid uptake, lipogenesis and lipolysis, are perturbed by extracellular stimuli, such as cytokines [38]. It is important to note that in some studies administration of TNF increases the lipolysis pathway and leads to increased concentration of free fatty acids in vivo and in vitro [38]. It is also important to note that TNF is known to cause a decrease in the expression of transporter of free fatty acid. Therefore, it may decrease free fatty acid uptake and induce hyperlipidaemia [38].

The parasites have the machinery for fatty acid synthesis (FAS II pathway). However, this pathway is not absolutely essential for the parasites in the blood stages [39]. Although FAS II appears to be non-essential in the blood stage of the parasites, in some patients infected with *Plasmodium* an upregulation of FAS II genes has been reported [40]. A detailed analysis in this respect is necessary to understand the mechanism(s) involved in lipid disequilibrium. In parasite extracts, hydroxyl derivatives of polyenoic fatty acids were found although they lack lipoxygenase enzyme, which is required for the formation of hydroxy fatty acids. Alternatively, hydroxy fatty acids could be generated by haem catabolism. Hydroxyacids play an inhibitory role in the monocyte function and hence mediate the host immune response. Lipid metabolism in parasites could therefore be an important part in host-parasite lipid balance [41]. Lipid metabolism is thus crucial, in many ways, for the survival of the parasite. It is also clear (Fig. 4) that as the disease progresses a number of peaks corresponding to lipids/lipoproteins appear in CM. This may be used as a qualitative identifier of CM in mice. The specific role of lipids in CM needs further investigation.

The changes in the lipid levels documented in this study are quantitative. The exact nature of enhanced lipids could not be resolved through these experiments. Quantitative mass spectrometric analysis may reveal specific lipid/phospholipid change in the CM mice. However, a threshold level may be able to be worked out for general lipid levels. In these studies, such a level fixation of fold change greater than 14 % can predict correctly the susceptibility for CM in about 54–71 % of the mice, independent of the sex of the animal. It may be possible to set such a threshold of lipids to predict susceptibility to CM versus other severity in malaria. This study can serve as a beginning for further investigation into the risk factors, mechanisms, vaccines, adjunct treatments for CM in the mouse model before the disease pathology sets in. It is proposed that the differences in serum lipids/lipoproteins of human patients be assessed so that a profile could be used as effective prognostic marker(s) of CM.

## Additional files

10.1186/s12936-016-1256-z PCA scores plot of CM vs NCM at day 4 post infection for experiment 4. The red and black symbols denote CM and NCM, respectively. The ellipse in the scores plot is a 95 % Hotelling T^2^.
10.1186/s12936-016-1256-z OPLS scores plot of CM and NCM of with ‘Y’ variables assigned as lipid/lipoprotein peaks in ^1^H NMR spectrum of the serum. The red and black symbols in the scores plot denote CM and NCM, respectively. (A) OPLS score plot of experiment 4. (B) OPLS scores plot of experiment 5.
10.1186/s12936-016-1256-z OPLS-DA scores and the coefficient plot of CM/NCM vs control of (a) male mice and (b) female mice of experiment 5. a – (A) OPSL-DA scores plot NCM and control, (B) OPLS-DA coefficient plot of (A), (C) OPLS-DA scores plot of CM and control, (D) OPLS-DA coefficient plot of (C). b – (A) OPSL-DA scores plot NCM and control, (B) OPLS-DA coefficient plot of (A), (C) OPLS-DA scores plot of CM and control, (D) OPLS-DA coefficient plot of (C). The red, black and blue symbols denote CM, NCM and control, respectively. The ellipse in the scores plot is a 95 % Hotelling T^2^. The colour bar indicates the correlation of the metabolites in the segregation between two concerned class.
10.1186/s12936-016-1256-z Parasitemia profile and the survival plot for CM and NCM mice. The open and the closed circles represent CM and NCM respectively.
10.1186/s12936-016-1256-z PCA scores analysis for CM and NCM at day 2, 3, 4, 5p.i. of Experiment 6. The red and the black symbols denote CM and NCM respectively.
10.1186/s12936-016-1256-z OPLS-DA scores and the coefficient plot of CM, NCM and control female mice at day 3 (a), day 4 (b) and day 5 (c) post infection of experiment 6. a – (A) OPSL-DA scores plot NCM and control, (B) OPLS-DA coefficient plot of (A), (C) OPLS-DA scores plot of CM and control, (D) OPLS-DA coefficient plot of (C). b – (A) OPSLDA scores plot NCM and control, (B) OPLS-DA coefficient plot of (A), (C) OPLS-DA scores plot of CM and control, (D) OPLS-DA coefficient plot of (C). c – (A) OPSL-DA scores plot NCM and control, (B) OPLS-DA coefficient plot of (A), (C) OPLS-DA scores plot of CM and control, (D) OPLS-DA coefficient plot of (C). The red, black and blue symbols denote CM, NCM and control, respectively. The ellipse in the scores plot is a 95 % Hotelling T^2^. The colour bar indicates the correlation of the metabolites in the segregation between two concerned class.
10.1186/s12936-016-1256-z Fold change of the normalized lipid peaks in the ^1^H NMR spectrum of CM mice with respect to controls at day 4 post infection.
10.1186/s12936-016-1256-z ROC plot of the OPLS-DA model of CM vs NCM females for three independent experiments at day 4 post infection (A) Experiment 4, (B) Experiment 5, (C) Experiment 6.

## Abbreviations

NMR: nuclear magnetic resonance
CM: cerebral malaria
NCM: non-cerebral malaria
pi: post-infection
OPLS-DA: orthogonal partial least square discriminant analysis
SHIRPA: SmithKline Beecham, Harwell, Imperial College, Royal London Hospital phenotype assessment
CPMG: Car–Purcell–Meiboom–Gill
ACQ: acquisition
COSY: correlated spectroscopy
TOCSY: total correlated spectroscopy
MSA: malaria score for adults
RBC: red blood cells
RD: relaxation delay
PCA: principal component analysis
GPC: glycerophosphocholine
HMDB: human metabolome database
ROC: receiver operating characteristic
