# Supplementary figures and images for: Early prediction of cerebral malaria by 1H NMR based metabolomics
Source: Malar J. 2016 Apr 12;15:198. doi: 10.1186/s12936-016-1256-z (PMC4828763; doi:10.1186/s12936-016-1256-z)

## Slide 1
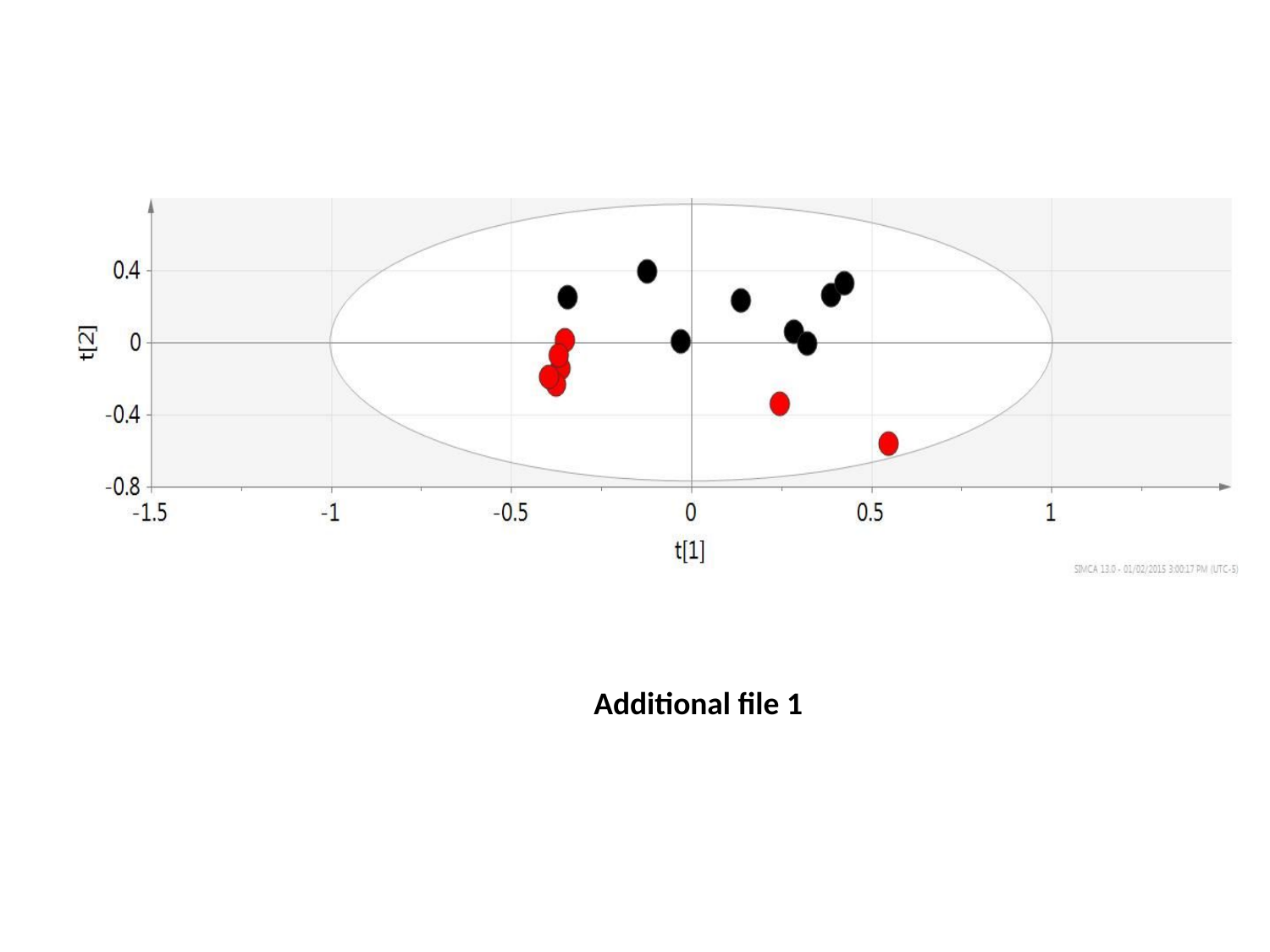

Additional file 1

Supplement: Supplementary file 1 — 10.1186/s12936-016-1256-z PCA scores plot of CM vs NCM at day 4 post infection for experiment 4. The red and black symbols denote CM and NCM, respectively. The ellipse in the scores plot is a 95 % Hotelling T2. [file 12936_2016_1256_MOESM1_ESM.pptx]

## Slide 1
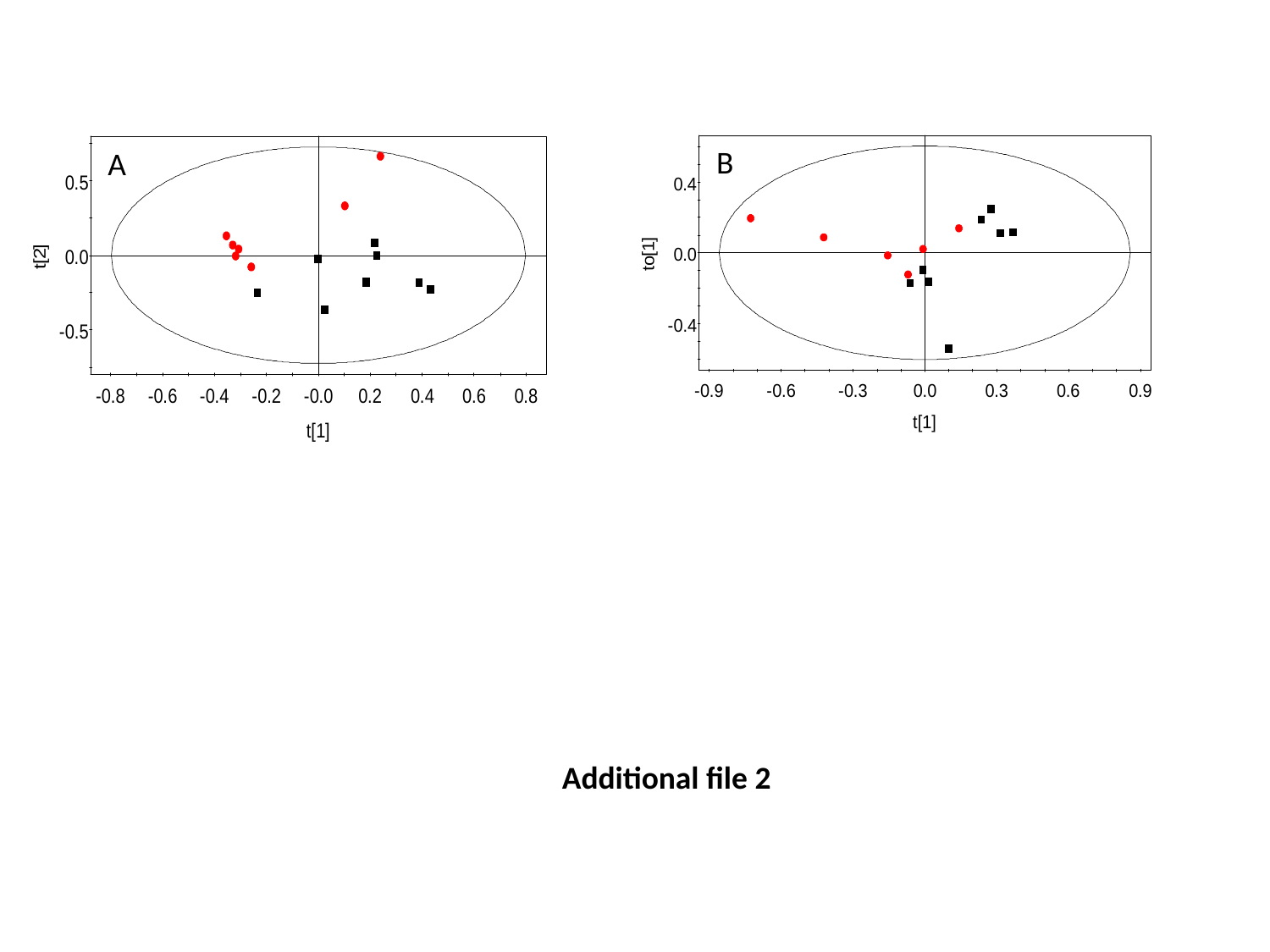

B
A
Additional file 2

Supplement: Supplementary file 2 — 10.1186/s12936-016-1256-z OPLS scores plot of CM and NCM of with ‘Y’ variables assigned as lipid/lipoprotein peaks in 1H NMR spectrum of the serum. The red and black symbols in the scores plot denote CM and NCM, respectively. (A) OPLS score plot of experiment 4. (B) OPLS scores plot of experiment 5. [file 12936_2016_1256_MOESM2_ESM.pptx]

## Slide 1
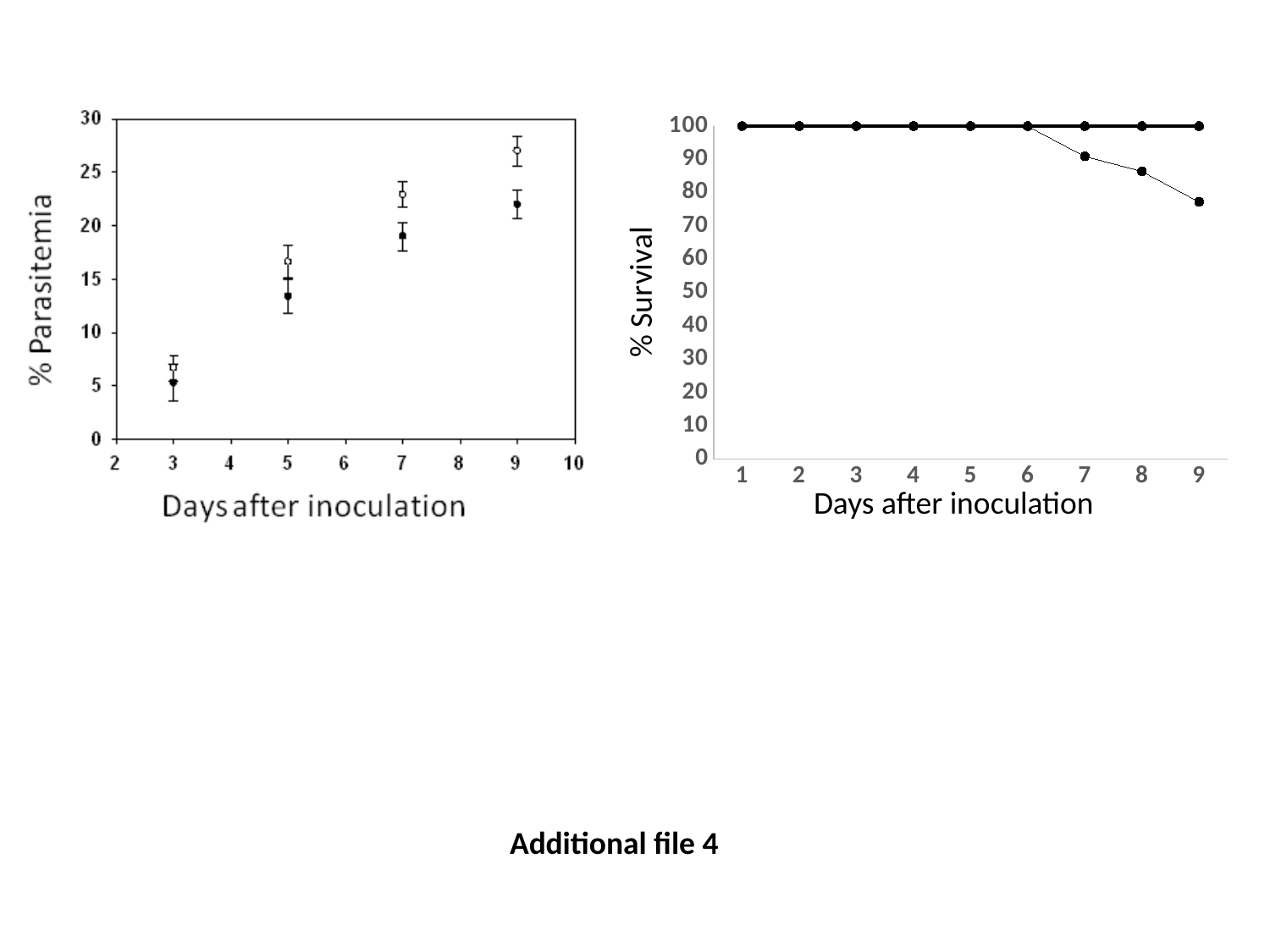

### Chart
| Category | | |
|---|---|---|
| 1 | 100.0 | 100.0 |
| 2 | 100.0 | 100.0 |
| 3 | 100.0 | 100.0 |
| 4 | 100.0 | 100.0 |
| 5 | 100.0 | 100.0 |
| 6 | 100.0 | 100.0 |
| 7 | 100.0 | 90.91000000000001 |
| 8 | 100.0 | 86.4 |
| 9 | 100.0 | 77.27272727272727 |% Survival
Days after inoculation
Additional file 4

Supplement: Supplementary file 4 — 10.1186/s12936-016-1256-z Parasitemia profile and the survival plot for CM and NCM mice. The open and the closed circles represent CM and NCM respectively. [file 12936_2016_1256_MOESM4_ESM.pptx]

## Slide 1
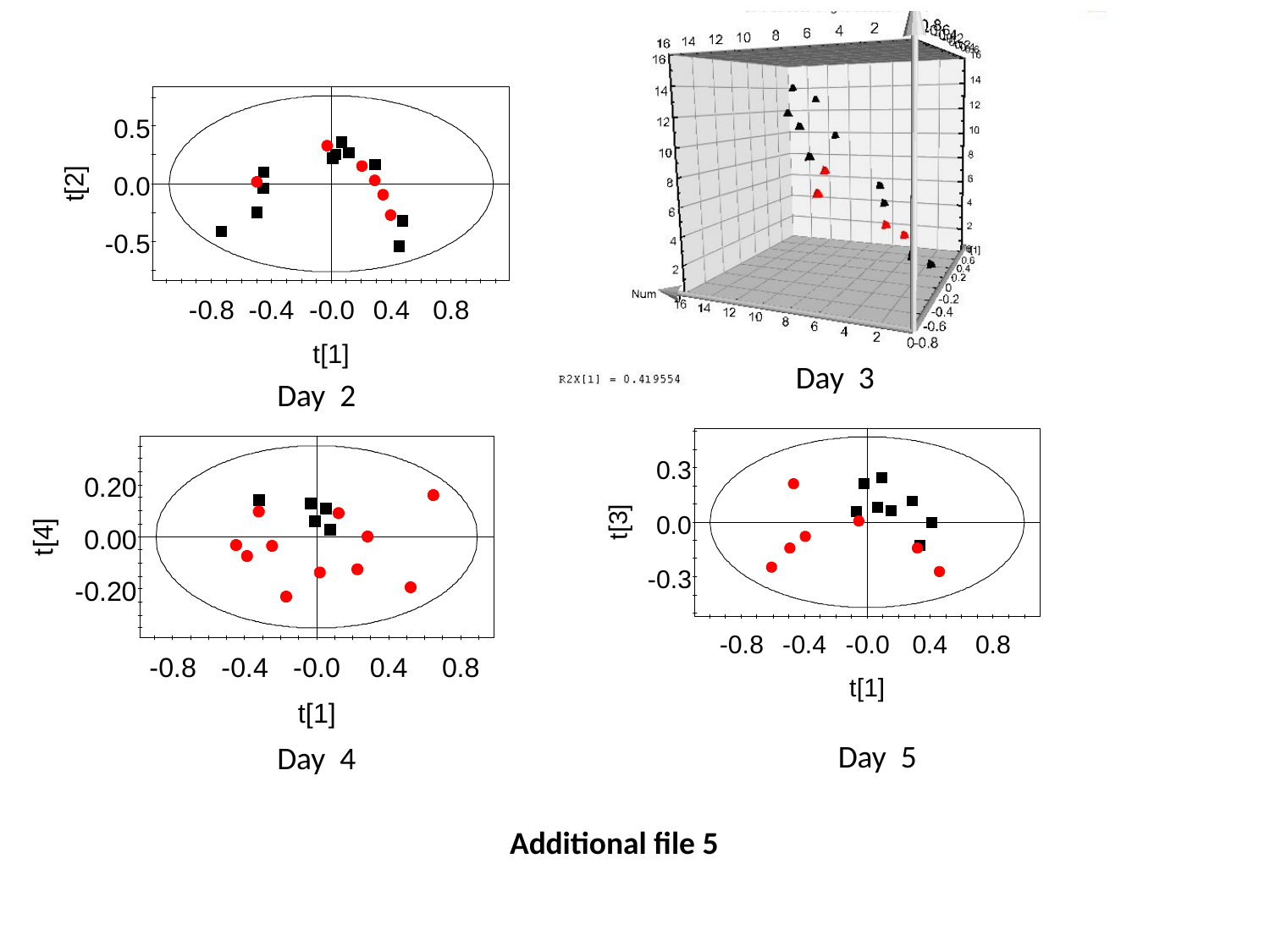

Day 3
Day 2
Day 5
Day 4
Additional file 5

Supplement: Supplementary file 5 — 10.1186/s12936-016-1256-z PCA scores analysis for CM and NCM at day 2, 3, 4, 5p.i. of Experiment 6. The red and the black symbols denote CM and NCM respectively. [file 12936_2016_1256_MOESM5_ESM.pptx]

## Slide 1
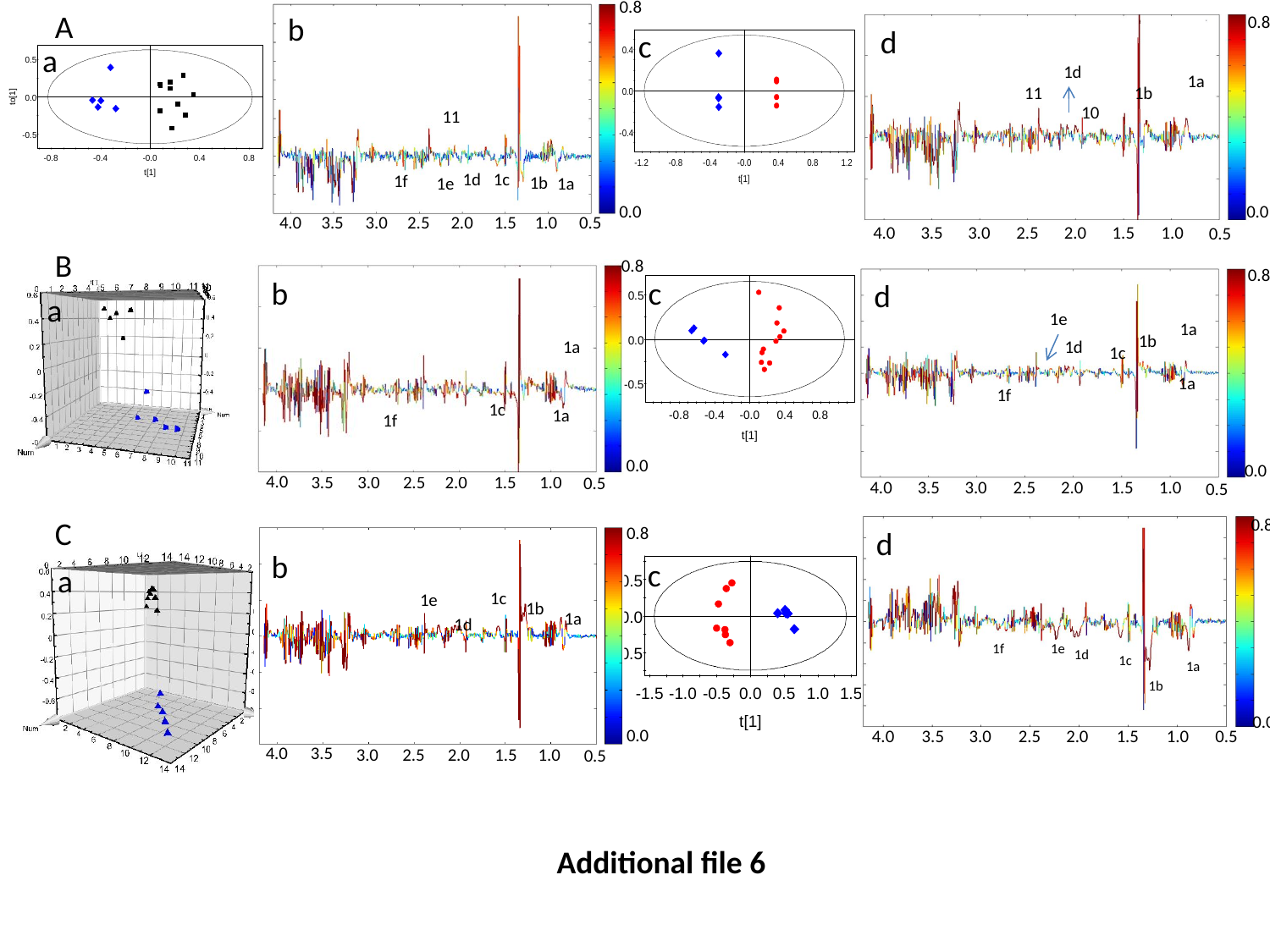

A
11
1c
1f
1b
1e
1a
4.0
1.5
1.0
0.5
0.8
b
0.8
d
c
a
1d
1a
11
1b
10
1d
1d
0.0
0.0
3.5
3.0
2.5
2.0
B
0.8
0.8
4.0
3.5
3.0
2.5
2.0
1.5
1.0
0.5
b
c
d
a
1e
1a
1b
1d
1a
1c
1d
1a
1f
1c
1c
1a
1f
1a
1f
0.0
0.0
0.8
C
1f
1e
1d
1c
1a
1b
d
1c
1e
1b
1a
1d
b
c
a
0.0
4.0
3.5
3.0
2.5
2.0
1.5
1.0
0.5
4.0
3.5
3.0
2.5
2.0
1.5
1.0
0.5
4.0
3.5
3.0
2.5
2.0
1.5
1.0
0.5
0.8
0.0
4.0
3.5
3.0
2.5
2.0
1.5
1.0
0.5
Additional file 6

Supplement: Supplementary file 6 — 10.1186/s12936-016-1256-z OPLS-DA scores and the coefficient plot of CM, NCM and control female mice at day 3 (a), day 4 (b) and day 5 (c) post infection of experiment 6. a – (A) OPSL-DA scores plot NCM and control, (B) OPLS-DA coefficient plot of (A), (C) OPLS-DA scores plot of CM and control, (D) OPLS-DA coefficient plot of (C). b – (A) OPSLDA scores plot NCM and control, (B) OPLS-DA coefficient plot of (A), (C) OPLS-DA scores plot of CM and control, (D) OPLS-DA coefficient plot of (C). c – (A) OPSL-DA scores plot NCM and control, (B) OPLS-DA coefficient plot of (A), (C) OPLS-DA scores plot of CM and control, (D) OPLS-DA coefficient plot of (C). The red, black and blue symbols denote CM, NCM and control, respectively. The ellipse in the scores plot is a 95 % Hotelling T2. The colour bar indicates the correlation of the metabolites in the segregation between two concerned class. [file 12936_2016_1256_MOESM6_ESM.pptx]
