# Supplementary material for: Early prediction of cerebral malaria by 1H NMR based metabolomics
Source: Malar J. 2016 Apr 12;15:198. doi: 10.1186/s12936-016-1256-z (PMC4828763; doi:10.1186/s12936-016-1256-z)
Supplement: Supplementary file 3 — 10.1186/s12936-016-1256-z OPLS-DA scores and the coefficient plot of CM/NCM vs control of (a) male mice and (b) female mice of experiment 5. a – (A) OPSL-DA scores plot NCM and control, (B) OPLS-DA coefficient plot of (A), (C) OPLS-DA scores plot of CM and control, (D) OPLS-DA coefficient plot of (C). b – (A) OPSL-DA scores plot NCM and control, (B) OPLS-DA coefficient plot of (A), (C) OPLS-DA scores plot of CM and control, (D) OPLS-DA coefficient plot of (C). The red, black and blue symbols denote CM, NCM and control, respectively. The ellipse in the scores plot is a 95 % Hotelling T2. The colour bar indicates the correlation of the metabolites in the segregation between two concerned class. [file 12936_2016_1256_MOESM3_ESM.pptx]

## Slide 1
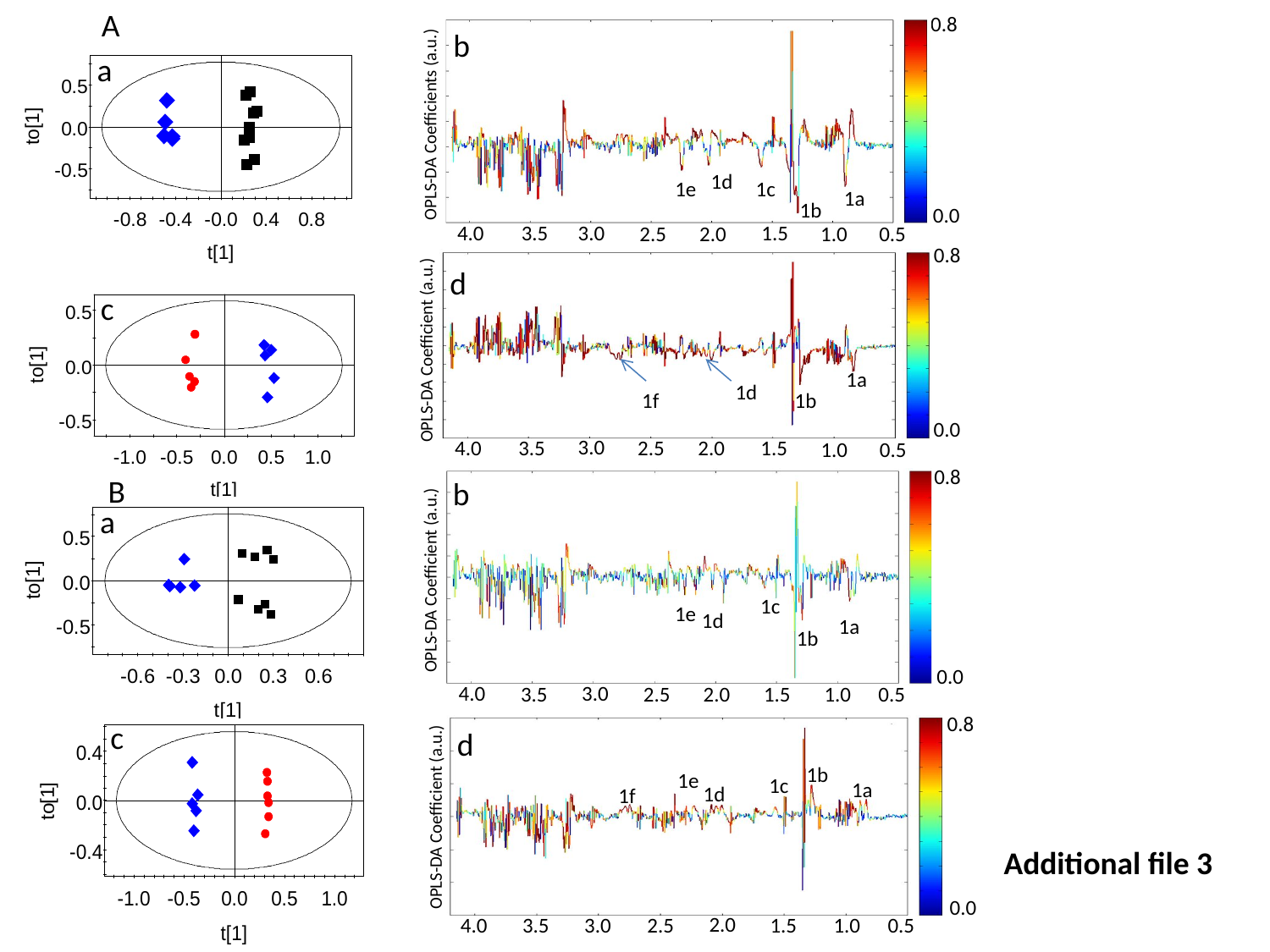

A
0.8
OPLS-DA Coefficients (a.u.)
1.5
3.5
4.0
3.0
2.5
0.5
2.0
1.0
1d
1e
1c
1a
1b
0.0
b
a
OPLS-DA Coefficient (a.u.)
1a
1d
1f
1b
3.0
2.5
3.5
1.5
4.0
2.0
1.0
0.5
0.8
0.0
d
c
0.8
b
OPLS-DA Coefficient (a.u.)
1c
1e
1a
1b
0.0
3.0
4.0
3.5
1.0
2.5
2.0
0.5
1.5
B
a
1d
0.8
0.0
2.0
4.0
3.5
3.0
2.5
1.5
1.0
0.5
d
OPLS-DA Coefficient (a.u.)
c
1b
1e
1c
1a
1d
1f
Additional file 3
