# Supplementary material for: Early prediction of cerebral malaria by 1H NMR based metabolomics
Source: Malar J. 2016 Apr 12;15:198. doi: 10.1186/s12936-016-1256-z (PMC4828763; doi:10.1186/s12936-016-1256-z)
Supplement: Supplementary file 7 — 10.1186/s12936-016-1256-z Fold change of the normalized lipid peaks in the 1H NMR spectrum of CM mice with respect to controls at day 4 post infection. [file 12936_2016_1256_MOESM7_ESM.pptx]

## Slide 1
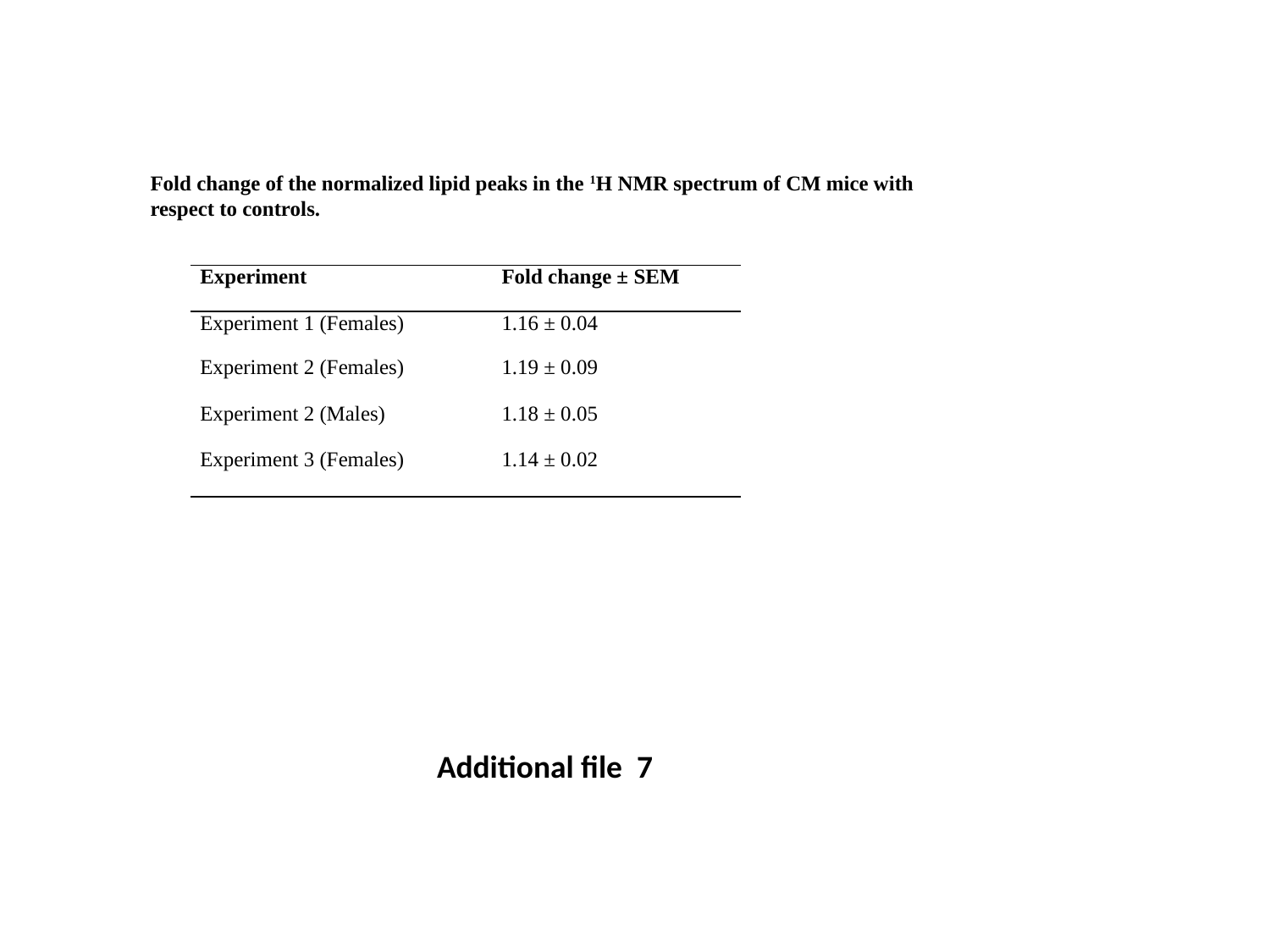

Fold change of the normalized lipid peaks in the 1H NMR spectrum of CM mice with respect to controls.
| Experiment | Fold change ± SEM |
| --- | --- |
| Experiment 1 (Females) | 1.16 ± 0.04 |
| Experiment 2 (Females) | 1.19 ± 0.09 |
| Experiment 2 (Males) | 1.18 ± 0.05 |
| Experiment 3 (Females) | 1.14 ± 0.02 |
Additional file 7
