# Supplementary material for: Early prediction of cerebral malaria by 1H NMR based metabolomics
Source: Malar J. 2016 Apr 12;15:198. doi: 10.1186/s12936-016-1256-z (PMC4828763; doi:10.1186/s12936-016-1256-z)
Supplement: Supplementary file 8 — 10.1186/s12936-016-1256-z ROC plot of the OPLS-DA model of CM vs NCM females for three independent experiments at day 4 post infection (A) Experiment 4, (B) Experiment 5, (C) Experiment 6. [file 12936_2016_1256_MOESM8_ESM.pptx]

## Slide 1
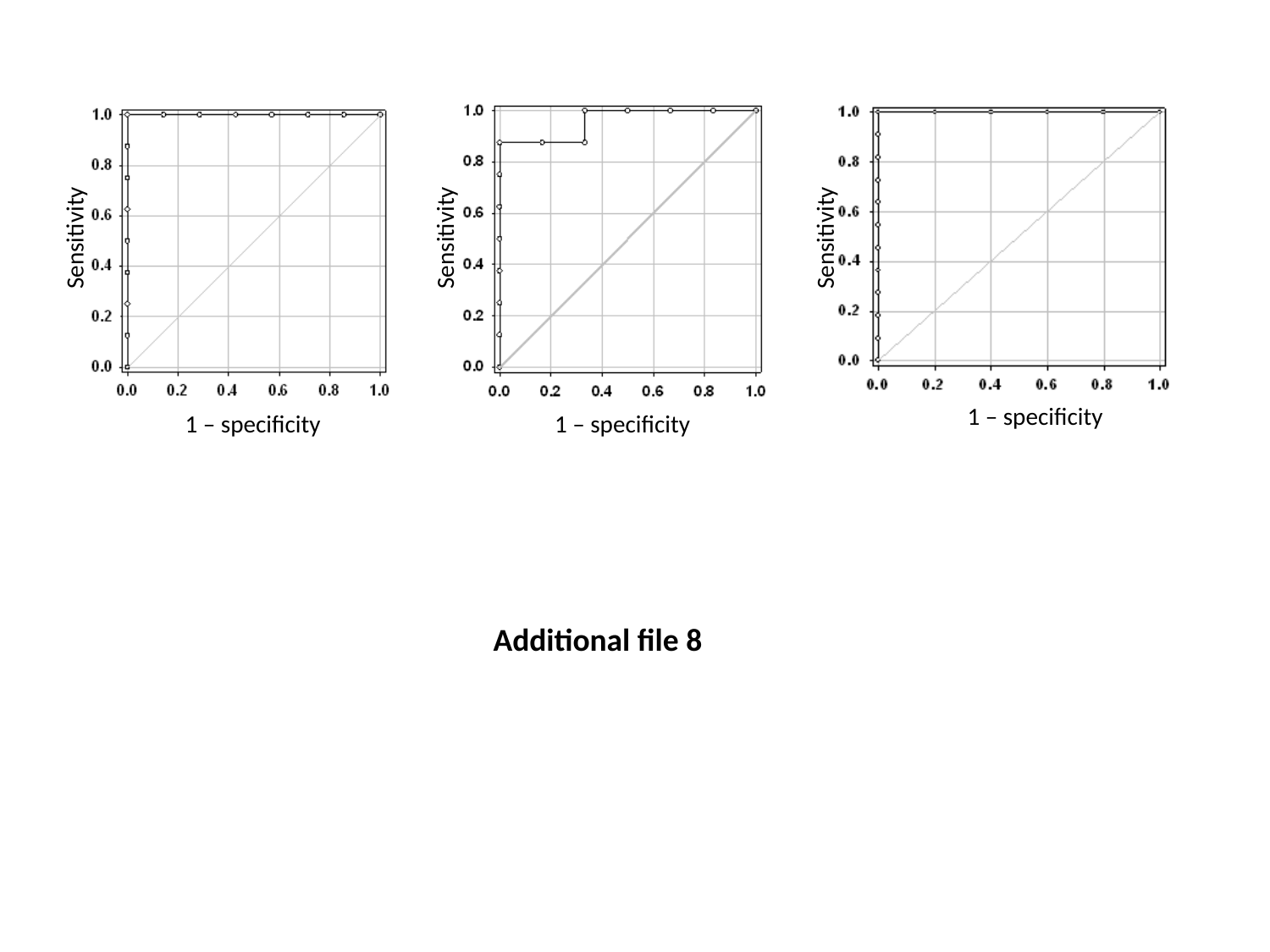

Sensitivity
Sensitivity
Sensitivity
1 – specificity
1 – specificity
1 – specificity
Additional file 8
